# True segmental lumbar imaging: Technical precision matters

**DOI:** 10.1016/j.inpm.2025.100641

**Published:** 2025-09-01

**Authors:** Vinil Shah

**Affiliations:** Department of Radiology & Biomedical Imaging, University of California San Francisco, 505 Parnassus Avenue, L-371, San Francisco, CA 94143, USA

Image-guided interventional pain procedures performed in appropriately selected patients in a technically precise manner would logically be expected to yield more beneficial outcomes and carry less risk than the same procedures performed otherwise.

Recent advances in interventional pain medicine techniques have improved technical successes and patient outcomes from established procedures. Cervical epidural access and lumbar medial branch radiofrequency neurotomy (LMBRFN) are two notable examples that come to mind. In 2015, Gill and colleagues demonstrated that contralateral oblique (CLO) imaging was superior to conventional lateral imaging for cervical interlaminar epidural access [1]. In 2020, Levi and colleagues showed that a modified approach using an MRI-measured superior articular process (SAP) angle trajectory avoided vulnerable neurovascular targets and provided superior epidural contrast medium flow compared to the conventional approach for cervical transforaminal epidural access [2]. These interlaminar and transforaminal technical improvements have advanced the safety and efficacy of these procedures and have been adopted [3].

Novel techniques for true AP imaging and true lateral lumbar spine imaging during LMBRFN have also recently been described [4,5]. As outlined in this current issue, the true AP imaging technique demonstrates near perfect interobserver agreement [6]. Likewise, similar levels of interobserver agreement have previously been shown for a true lateral imaging technique [7].

True AP and true lateral imaging, when utilized together, form true segmental imaging [6]. True segmental imaging establishes the location of a specific-segment RF cannula in relation to the bony target for medial branch coagulation [8]. Conversely, without true imaging, the proceduralist cannot be certain about the location of the RF lesion in relation to the targeted medial branch [8]. The use of true segmental imaging during other lumbar spine interventions has also been advised [2].

Systematic reviews inform physicians and policy-makers regarding the efficacy and risks of specific procedures. If a particular procedure's outcome studies are based on a sub-optimal technique, procedural outcomes are likewise adversely impacted. Conversely, employing advances in technical precision during procedures enhances efficacy and leads to improved outcome studies and greater support in future systematic reviews. As demonstrated in this issue, true segmental imaging provides a validated approach to guide and determine needle/device placement in the lumbar spine and represents another important advance in interventional pain medicine.

## Declaration of competing interest

The authors declare the following financial interests/personal relationships which may be considered as potential competing interests: Vinil Shah reports a relationship with International Pain & Spine Intervention Society that includes: board membership and travel reimbursement. If there are other authors, they declare that they have no known competing financial interests or personal relationships that could have appeared to influence the work reported in this paper.
